# Metabolic Control of Germline Formation and Differentiation in Mammals

**DOI:** 10.1159/000520662

**Published:** 2022-01-27

**Authors:** Yohei Hayashi, Yasuhisa Matsui

**Affiliations:** ^a^Cell Resource Center for Biomedical Research, Institute of Development, Aging and Cancer (IDAC), Tohoku University, Sendai, Japan; ^b^Graduate School of Life Sciences, Tohoku University, Sendai, Japan; ^c^Graduate School of Medicine, Tohoku University, Sendai, Japan

**Keywords:** Development, Differentiation, Epigenome, Germ cells, Metabolism

## Abstract

**Background:**

The germ cell lineage involves dynamic epigenetic changes during its formation and differentiation that are completely different from those of the somatic cell lineage. Metabolites and metabolic pathways have been reported as key factors related to the regulation of epigenetics as cofactors and substrates. However, our knowledge about the metabolic characteristics of germ cells, especially during the fetal stage, and their transition during differentiation is quite limited due to the rarity of the cells. Nevertheless, recent developments in omics technologies have made it possible to extract comprehensive metabolomic features of germ cells.

**Summary:**

In this review, we present the latest researches on the metabolic properties of germ cells in 4 stages: primordial germ cell specification, fetal germ cell differentiation, spermatogenesis, and oogenesis. At every stage, extensive published data has been accumulated on energy metabolism, and it is possible to describe its changes during germ cell differentiation in detail. As pluripotent stem cells differentiate into germ cells, energy metabolism shifts from glycolysis to oxidative phosphorylation; however, in spermatogenesis, glycolytic pathways are also temporarily dominant in spermatogonial stem cells. Although the significance of metabolic pathways other than energy metabolism in germ cell differentiation is largely unknown, the relation of the pentose phosphate pathway and Ser-Gly-one-carbon metabolism with germ cell properties has been suggested at various stages. We further discuss the relationship between these characteristic metabolic pathways and epigenetic regulation during germ cell specification and differentiation. Finally, the relevance of dietary and supplemental interventions on germ cell function and epigenomic regulation is also discussed.

**Key Messages:**

Comprehensive elucidation of metabolic features and metabolism-epigenome crosstalk in germ cells is important to reveal how the characteristic metabolic pathways are involved in the germ cell regulation. The accumulation of such insights would lead to suggestions for optimal diets and supplements to maintain reproductive health through modulating metabolic and epigenetic status of germ cells.

## Introduction

**Fig. 1:**
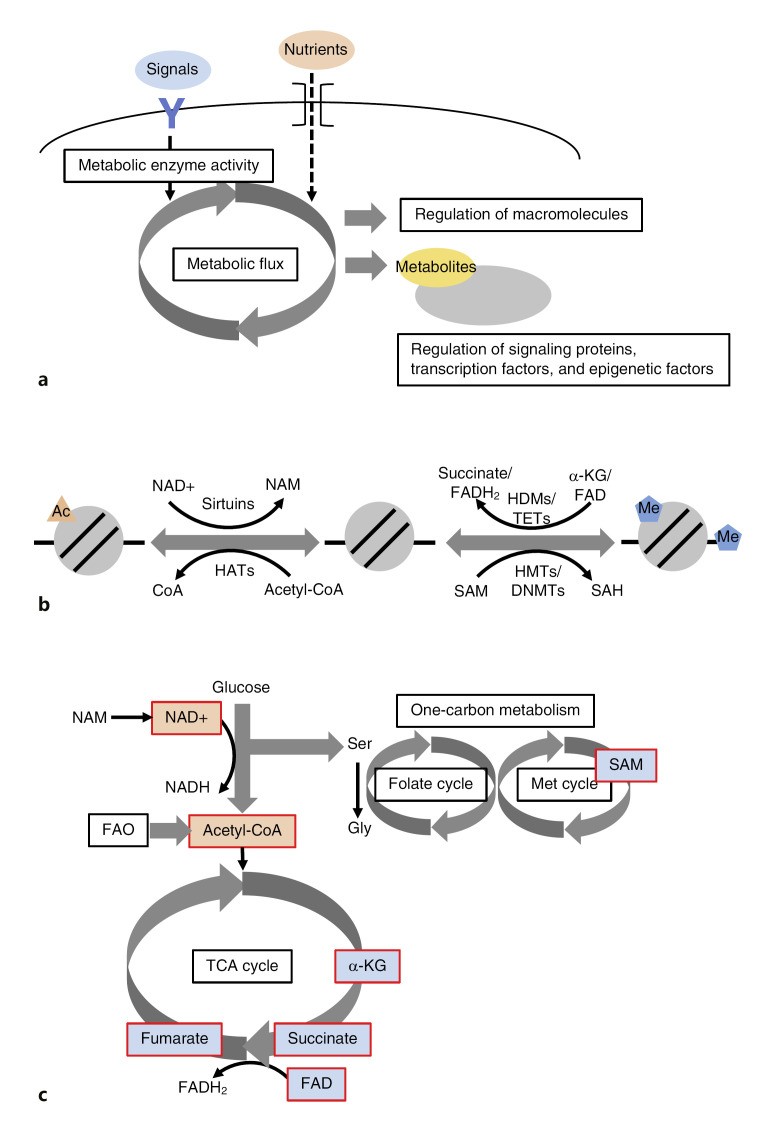
**a** Schematic view of the link between metabolic changes and cellular function. **b** Epigenetic reactions via chromatin-modifying enzymes, their substrates, and co-factors. **c** Metabolic pathways related to epigenome-related metabolites. Metabolites related to histone acetylation and histone/DNA methylation are shaded in orange and blue, respectively. α-KG, α-keto­glutarate; CoA, coenzyme A; DNMT, DNA methyltransferase; FAD, flavin adenine dinucleotide; FAO, fatty acid oxidation; HAT, histone acetyltransferase; HDM, histone demethylase; HMT, histone methyltransferase; NAD+, nicotinamide adenine dinucleotide; NAM, nicotinamide; SAH, S-adenosyl homocysteine; SAM, S-adenosyl methionine; TCA, tricarboxylic acid; TET, ten-eleven translocation DNA demethylase.

Changes in cellular state and function, including differentiation of stem and progenitor cells into various cell types, are accompanied by extensive rearrangements in metabolic demands and features through the changes in extracellular environments [Agathocleous and Harris, 2013] (Fig. 1a). In the regulation of differentiation, cell-extrinsic and -intrinsic signals such as cytokines and transcription factors, respectively, could lead to changes in metabolic enzyme expression and activity, which cause a fundamental shift of metabolic flux. In addition, changes in the nutritional status of the extracellular environment could also cause a shift of metabolic flux. Such changes in metabolic flux are linked to the alteration in metabolic demands and affect the syntheses of structural and functional components of the cells. Furthermore, changes in metabolic flux could also regulate the concentration of specific metabolites, which regulate signaling pathways and gene expression and consequently influence cell differentiation. In this context, metabolism is also believed to be a principal regulator of the epigenome, chemical modifications of histones and DNA, as most chromatin-modifying enzymes utilize particular metabolites as substrates or cofactors to support their catalytic activities [Haws et al., 2020].

This metabolism-epigenome crosstalk appears to be quite complex in which metabolic cofactors and enzymes dynamically regulate various types of the epigenome, including acetylation, methylation of histones, and 5-methylcytosine (5mC) DNA methylation [Haws et al., 2020] (Fig. 1b). For instance, histone acetyltransferases (HATs) require acetyl-coenzyme A (CoA) as the acetyl-donating substrate [Sabari et al., 2017], while class III histone deacetylases (HDACs), also known as Sirtuins (SIRTs), utilize a nicotinamide adenine dinucleotide (NAD+) molecule during catalysis [Feldman et al., 2012]. In the case of methylation, both histone methyltransferases (HMTs) and DNA methyltransferases (DNMTs) require S-adenosylmethionine (SAM) as the methyl-donor substrate. On the other hand, a class of histone demethylases (HDMs), lysine-specific demethylases (LSDs), utilize flavin adenine dinucleotide (FAD) for the oxidation of the methylated lysine [Shi et al., 2004]. Another class of HDMs, jumonji C (JmjC) domain-containing demethylases, and a family of DNA demethylases, TETs (ten-eleven translocation DNA demethylase), which convert 5mC to 5-hydroxymethylcytosine (5hmC) [Ito et al., 2011], progress lysine/arginine and 5mC demethylation, respectively, in the same way using α-ketoglutarate (α-KG) in dioxygenase reactions [Anand and Marmorstein, 2007; Wu and Zhang, 2017]. In contrast, succinate and structurally similar fumarate can inhibit activities of these demethylases [Xiao et al., 2012].

The level of these epigenome-related metabolites is regulated by various metabolic pathways [Haws et al., 2020] (Fig. 1c). Related to histone acetylation, acetyl-CoA can be generated by the pyruvate dehydrogenase complex (PDC) in mitochondria and through metabolic pathways such as fatty acid oxidation (FAO) [Fan et al., 2015]. For epigenetic regulation, mitochondrial acetyl-CoA must first be converted to citrate to be exported to the cytoplasm, and citrate is converted back to acetyl-CoA in the nucleus [Sivanand et al., 2018]. A Sitruin co-factor, NAD+, is synthesized mainly through the nicotinamide (NAM) salvage pathway and the NADH oxidation in the electron transport chain [Verdin, 2015]. The NAD+/NADH ratio has been reported to critically regulate the activities of SIRTs [Chakrabarty and Chandel, 2021]. As for the histone/DNA methylation-related metabolites, SAM is generated as a product of ATP-dependent methionine adenosyltransferase (MAT) reactions in one-carbon metabolism consisting of the folate and methionine cycles in mammalian cells [Sanderson et al., 2019]. FAD is an electron acceptor for the tricarboxylic acid (TCA) cycle enzyme succinate dehydrogenase, producing FADH_2_, which is used by the electron transport chain to drive ATP synthesis [Haws et al., 2020]. Metabolites that regulate the activities of HDMs/TETs, α-KG, succinate, and fumarate are the TCA cycle intermediates. Succinate is also produced from structurally similar α-KG by the JmjC reaction, so that succinate can competitively inhibit JmjC catalysis against α-KG by the negative feedback [Xiao et al., 2012]. This indicates that change in the α-KG/succinate ratio is capable of directly influencing histone and DNA demethylation and subsequent gene expression [Carey et al., 2015].

Due to the requirement of metabolic cofactors by many chromatin modifying enzymes as described above, the epigenome is susceptible to perturbations in such cellular metabolisms [Haws et al., 2020]. For example, the abundance of various metabolites in the liver is regulated by circadian mechanisms, including co-factors for chromatin modifying enzymes (e.g., SAM, NAD+, and FAD) [Krishnaiah et al., 2017]. Circadian oscillation of NAD+ biosynthesis, an essential cofactor for the deacetylase SIRT1, affects the acetylation status of histone H3 lysine 9 (H3K9ac) and lysine 14 (H3K14ac) controlled by the CLOCK-BMAL1 HAT complex and SIRT1, becoming highly acetylated during the low NAD+ period that results in the upregulation of circadian-regulated genes [Doi et al., 2006; Nakahata et al., 2008]. Moreover, acetyl-CoA has been reported to increase with age in *Drosophila*, which promotes significant increases in histone acetylation and age-associated gene expression [Peleg et al., 2016]. In contrast, the age-dependent decrease in NAD+ diminishes HDAC activity of SIRTs and leads to global increases in histone acetylation levels, thereby compromising heterochromatin stability [Dang et al., 2009]. Furthermore, tumor cells are well known to undergo reprogramming of energy metabolism, highlighted by the presence of the Warburg effect, a phenomenon in which cancer cells produce ATP in glycolysis rather than mitochondrial oxidative phosphorylation (OXPHOS) even under aerobic conditions [Warburg, 1956]. A consequence of the Warburg effect is elevated intracellular concentrations of acetyl-CoA, resulting in the alteration of histone acetylation patterns seen in many solid tumors [Liberti and Locasale, 2016]. In addition to histone acetylation, histone and DNA methylation are also linked to altered cancer metabolism. Mutations in isocitrate dehydrogenases IDH1 and IDH2, which normally catalyze the conversion of isocitrate to α-KG in the TCA cycle, change their enzymatic activity to catalyze the reduction of α-KG to 2-hydroxyglutarate (2-HG), which functions as a competitive inhibitor for JmjC and TET demethylases and causes genome-wide alterations of these methylation patterns [Xu et al., 2011]. Loss of function mutations to succinate and fumarate dehydrogenases are also associated with various cancers, resulting in the accumulation of succinate and fumarate, respectively, which are capable of inhibiting JmjC and TET demethylases [Xiao et al., 2012].

Among the various cell types that constitute an animal body, germ cells in particular undergo dynamic epigenetic changes to differentiate into gametes and acquire totipotency [Sasaki and Matsui, 2008; Matsui and Mochizuki, 2014]. Though germ cells go through a series of unique epigenetic events that results in a unique gene-expression program that is different from somatic cells, the relationship of metabolism to the dynamic epigenetic changes during germ cell development has not been fully elucidated, due to the paucity of studies related to germ cell metabolism, especially in the fetal stage. Although limited amounts of germ cells were an obstacle for the comprehensive investigation of metabolomic dynamics, recent progress in metabolomic and proteomic analyses has made it possible to comprehensively reveal the metabolic characteristics of germ cells during specification and differentiation. In this review, we cover our current understanding of what and how metabolism dynamically regulates germ cell formation and development. We highlight the comprehensive metabolic changes from germ cell specification and differentiation in fetal stage to gametogenesis in mammals. In addition, we discuss the known and potential links of metabolism with the epigenome and their biological significance during germline formation and differentiation. Since metabolism in spermatogenesis and oogenesis has been systematically reviewed by several authors [Boussouar and Benahmed, 2004; Thompson, 2006; Sutton-McDowall et al., 2010; Rato et al., 2012; Gu et al., 2015; Schagdarsurengin and Steger, 2016], brief summaries of metabolic characteristics and discussions for the relationship between metabolism and the epigenome in gametogenesis are presented in this review.

## Metabolism in Germ Cell Specification

**Fig. 2:**
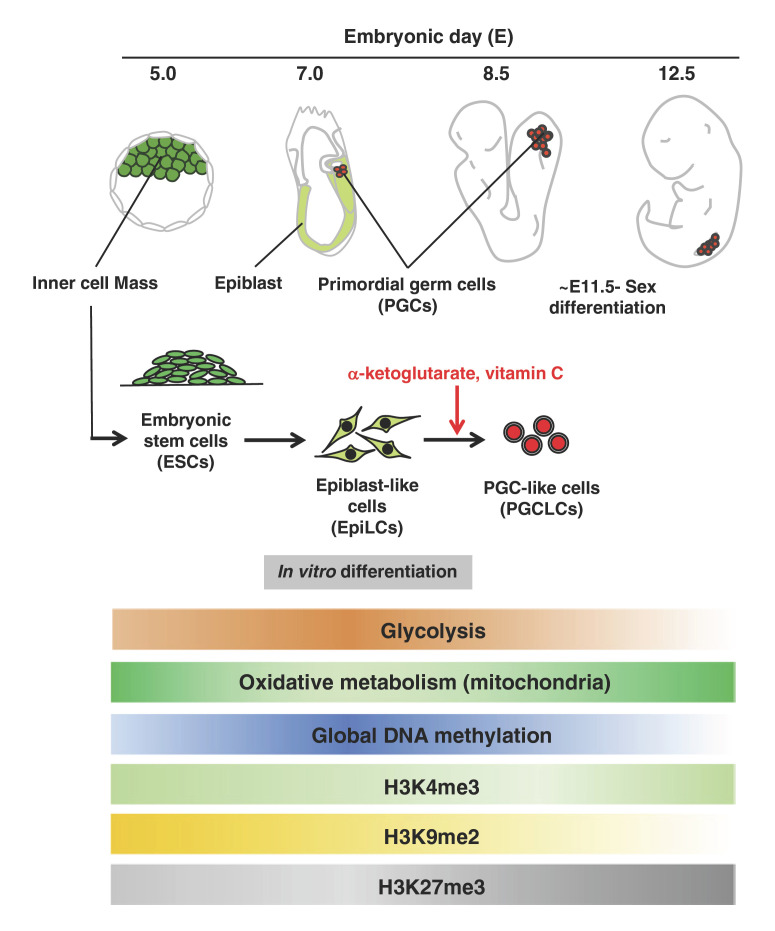
Global changes in metabolism and epigenome during mouse PGC specification and early PGC differentiation from E5.0 to E12.5 are indicated. The shading of the bars represents the predicted activation state of each metabolic pathway, with darker bars indicating higher activity. The naïve and primed stem cells are highlighted with green and light green in the embryos, respectively, while PGCs are highlighted with red. The red text shows the metabolic pathways or metabolites that affect the specification or differentiation of PGCs.

Embryonic stem cells (ESCs) derived from the inner cell mass (ICM) of murine blastocysts are considered the developmental naïve state that gives rise to all somatic and germline lineages, and utilizes bivalent metabolism (both glycolysis and OXPHOS) [Nichols and Smith, 2009; Weinberger et al., 2016; Tsogtbaatar et al., 2020]. In contrast, post-implantation epiblast cells are predominantly glycolytic [Weinberger et al., 2016]. Single cell RNA-seq of ESCs in the pluripotent ground state 2i/LIF (leukemia inhibitory factor) culture conditions and primed epiblast-like cells revealed a switch to an increased glycolytic state with a concomitant decrease in oxidative metabolism [Ying et al., 2008; Tischler et al., 2019] (Fig. 2). In post-implantation mammalian embryos, a population of pluripotent stem cells in the epiblast gives rise to primordial germ cells (PGCs), the precursors of both spermatozoa and oocytes. In mice, PGCs first emerge inside the extra-embryonic mesoderm at the posterior end of the primitive streak as a cluster of cells at embryonic day 7.25 (E7.25) [Ginsburg et al., 1990; Saitou and Yamaji, 2012]. To elucidate the metabolic characteristics and their alteration from PGC specification to male gonadal germ cell (gonocyte) formation in mice, we previously performed energy metabolism analyses of ESCs, PGC-like cells (PGCLCs) induced from epiblast-like cells (EpiLCs) *in vitro*, which is known to be equivalent to about E9.5 PGCs [Hayashi et al., 2011], E13.5 male gonocytes, and gonadal somatic cells (Somas) [Hayashi et al., 2017]. Our results demonstrated that sequential conversion of the major energy metabolic pathway from glycolysis to OXPHOS occurs specifically in germ cells during differentiation and that this conversion plays an important role in reprogramming, survival, and specification of PGCs from pluripotent stem cells [Hayashi et al., 2017]. A further study compared the metabolic features of EpiLCs and PGCLCs by untargeted metabolomics analysis and revealed an increased pyruvate/lactate ratio and elevated levels of TCA cycle intermediates and glutamate in PGCLCs [Xing et al., 2020]. All these findings strongly suggest that according to the formation of PGCs, cellular energy metabolism switches from glycolysis to aerobic respiration in mitochondria (Fig. 2).

PGCs have been reported to have relatively few numbers of mitochondria compared with ESCs at an early stage, indicating the restricted mitochondrial activity in PGCs immediately after formation [Floros et al., 2018]. In the study, single cell deep mitochondrial DNA (mtDNA) sequencing of *in vivo* mouse and human female PGCs showed rare variants at an early stage and higher heteroplasmy levels as a result of a massive proliferation of mtDNA molecules within the migrating PGCs. This indicated a mitochondrial genetic bottleneck during PGC specification, in which a selected number of mtDNA molecules are transferred into the germline and then amplified during PGC proliferation. The genetic bottleneck leads to a random distribution of mutated mtDNA between each germ cell. In the case that the mtDNA molecules have deleterious mutations, the energy metabolic activity and/or mtDNA replication of the cells would be adversely affected, resulting in the removal through negative selection. Therefore, the energy metabolic activity of PGCs may be involved in the selection process of healthy germ cells through a mitochondrial genetic bottleneck during PGC specification.

PGC specification is accompanied by epigenetic remodeling, including large-scale reorganization of chromatin signatures like H3K4 trimethylation (me3), H3K27me3, H3K9 dimethylation (me2), and 5mC demethylation [Seki et al., 2005; Kurimoto et al., 2015; Shirane et al., 2016] (Fig. 2). The studies using PGCLC induction have reported that during PGCLC induction, H3K4me3 is lost from many bivalent genes, which have both activating H3K4me3 and repressing H3K27me3 marks and are prevalent in developmental genes, while H3K27me3 is rather upregulated specifically around transcriptional start sites (TSSs), indicating the strong repression of developmental regulators for unrelated lineages to the germline [Kurimoto et al., 2015]. Also, cells progressively lose H3K9me2 during PGCLC induction from pluripotent stem cells, including at lamina-associated domains, resulting in changes in nuclear architecture. On the other hand, global elevation of 5mC DNA methylation was reported to occur during ESC-to-EpiLC transition, while genome-wide 5mC demethylation was caused during PGCLC induction from EpiLCs [Shirane et al., 2016]. PGCLCs have demethylation-sensitive domains around developmental genes which are repressed through abundant H3K27me3. Due to such functional importance of epigenetic regulation, epigenetics-related metabolites tend to be functional during PGC specification. A recent study using the induction of mouse PGCLCs from EpiLCs demonstrated that an intermediate metabolite of the TCA cycle, α-KG, maintains the developmental competence of EpiLCs for the PGC fate by preventing EpiLCs from attaining the high H3K9me2 and low H3K27me3 levels [Tischler et al., 2019]. In addition, it has been found that intake of essential micronutrients also affects PGC specification. A study using human PGCLCs [Irie et al., 2015] also suggested that supplementation with vitamin C, which enhance the activities of JmjC demethylases and TET proteins [Lee Chong et al., 2019], increased the levels of 5hmC and improved the expression of key genes involved in early germ cell development such as *NANOS3*, *TFAP2C*, *BLIMP1*, and *SOX17* [Li et al., 2019]. These studies suggest that adjustment of epigenetics by both intracellular metabolites and external micronutrients influences PGC specification (Fig. 2).

Taken together, all these studies suggest the importance of a shift in energy metabolism and metabolism-related epigenetic remodeling during PGC specification [Verdict and Allard, 2021]. Further comprehensive analysis of metabolic transitions should reveal the biological significance of metabolic regulation during PGC specification. In addition, the coordination of metabolism and the epigenome during PGC specification is also a very interesting issue. For example, 5mC DNA methylation and the glycolytic activity showed similar changes during PGC specification, becoming increased in ESC-to-EpiLC transition and then decreased in PGCLC induction from EpiLCs, and these changes were inversely correlated with the changes in the OXPHOS activity [Shirane et al., 2016; Tischler et al., 2019] (Fig. 2). Since an intermediate of the TCA cycle, α-KG, positively regulates DNA demethylation, a shift of the energy metabolism may work as an upstream regulator of the DNA methylation state. Furthermore, the timing of the switch from glycolysis to oxidative metabolism and that of the changes in histone methylation, such as H3K4me3 and H3K27me3 in promoter regions, appear to correspond well in EpiLC-to-PGCLC transition [Kurimoto et al., 2015]. Such dynamic changes in histone methylation may involve the dynamic regulation of the HMT and HDM activities, indicating that the metabolic regulation related to these enzymes via intermediates in the TCA cycle and SAM supply through one-carbon metabolism may affect PGC specification.

## Metabolism in Fetal Germ Cell Differentiation

After specification, PGCs undergo proliferation and migrate to the developing hindgut endoderm at about E7.75, into the mesentery at about E9.5, and colonize the genital ridges at about E10.5 [Saitou and Yamaji, 2012]. During PGC migration and proliferation, levels of genome-wide epigenetic modifications dynamically change, including the decrease of H3K9me2 and 5mC methylation, and the upregulation of H3K27me3 [Seki et al., 2005, 2007]. After they arrive at the genital ridge around E10.5, PGCs undergo the global erasure of pre-existing 5mC DNA methylation including the elimination of parental imprints, epigenetic marks that ensure parental-origin-derived monoallelic expression of imprinted genes in the next generation, and establishes new epigenetic patterns [Seisenberger et al., 2012]. After sex determination in germ cells around E11.5, male gonocytes enter into mitotic arrest in the G0/G1 phase around E14.5-E15.5. In male embryos, genome-wide DNA re-methylation occurs in the gonocytes and is completed before birth. After that, these cells resume active proliferation around day 5 postpartum (P5), and some of them develop into spermatogonial stem cells (SSCs) and differentiating spermatogonia, followed by meiotic entry [Spradling et al., 2011]. In comparison, female germ cells enter into meiosis around E13.5 and are then arrested in prophase I (diplotene stage) in the neonatal ovaries. Simultaneously, female gonadal germ cells form germ cell cysts from E10.5 to E14.5 [Lei and Spradling, 2013], leading to the formation of ovigerous cords consisting of germ cell cysts and their surrounding pre-granulosa cells by the perinatal stage [Suzuki et al., 2015]. After birth, the germ cell nests break down into single oocytes surrounded by granulosa cells, resulting in the assembly of primordial follicles. DNA is almost completely unmethylated in non-growing oocytes, and H3K4me3 and H3K27me3 are exclusively enriched at active promoters and broad domains including promoters and intergenic regions, respectively [Tomizawa et al., 2012; Hanna et al., 2018]. By the fully-grown germinal vesicle (GV) stage, DNA across transcribed gene bodies is fully methylated and H3K4me3 is accumulated in broad untranscribed regions.

**Fig. 3:**
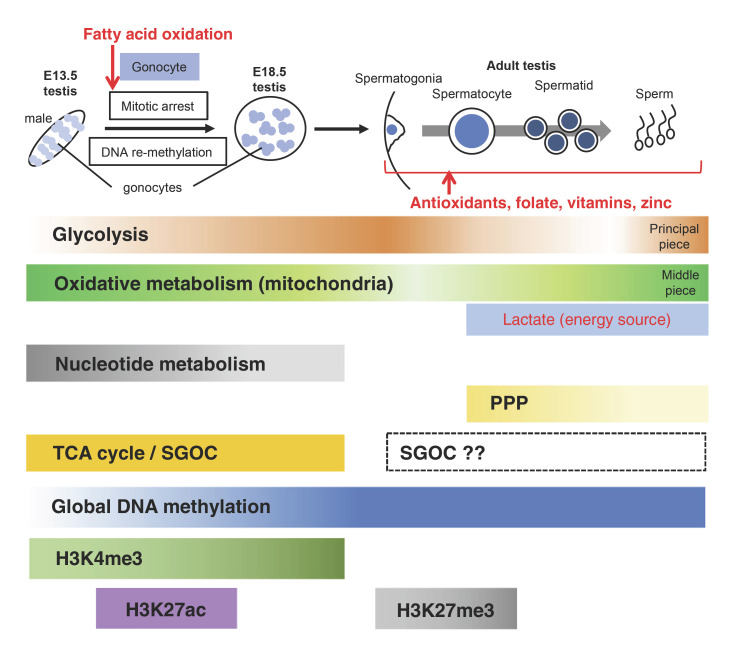
Global changes in metabolism and epigenome during fetal male germ cell differentiation and spermatogenesis. The shading of the bars represents the predicted activation state of each metabolic pathway, with darker bars indicating higher activity. The question marks (?) indicate putative relation, as described in the text. The red text shows the metabolic pathways or metabolites that affect each process in male germ cell differentiation. PPP, pentose phosphate pathway; SGOC, Ser-Gly-one-carbon metabolism.

**Fig. 4:**
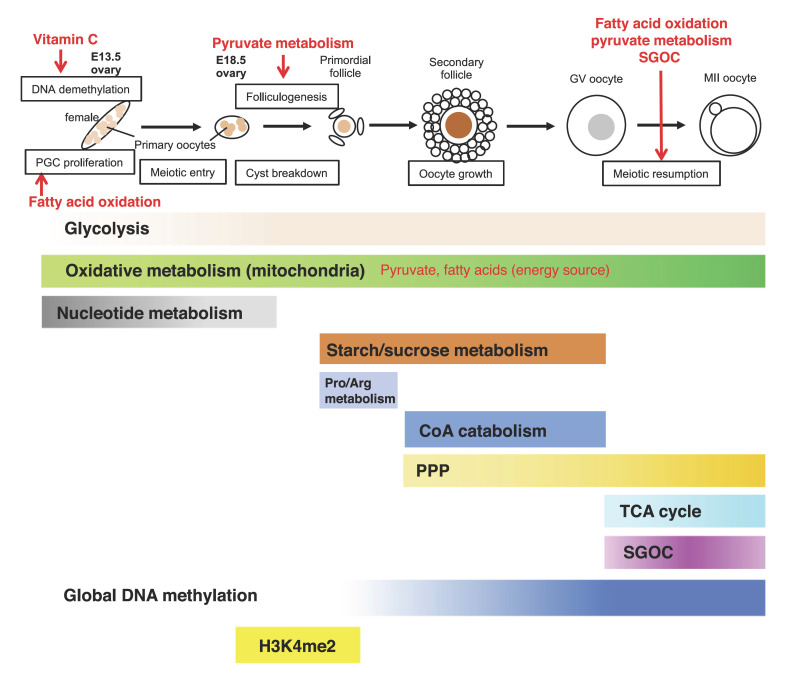
Global changes in metabolism and epigenome during fetal female germ cell differentiation, oocyte growth, and maturation. The shading of the bars represents the predicted activation state of each metabolic pathway, with darker bars indicating higher activity. The red text shows the metabolic pathways or metabolites that affect each process in female germ cell differentiation. PPP, pentose phosphate pathway; SGOC, Ser-Gly-one-carbon metabolism.

In spite of such intensive epigenetic changes during fetal germ cell differentiation, there are only limited studies on metabolic regulation of fetal germ cell differentiation, especially during PGC migration. The metabolic state of fetal gonadal germ cells in mice was first measured in 1977, showing the predominance of aerobic respiration and low glycolytic activity in E15 fetal germ cells compared with fertilized eggs [Brinster and Harstad, 1977]. In humans, mitochondrial copy number per germ cells was reported to be the fewest in pre-migratory PGCs, then gradually increased during germ cell differentiation, indicating the increasing activity of mitochondrial metabolism [Jansen and de Boer, 1998]. We have recently elucidated the changes of energy metabolism during fetal germ cell differentiation [Hayashi et al., 2020; Tanaka et al., 2021]. Although the results were consistent with the previous study that E13.5 germ cells exhibited very low glycolytic activity and high OXPHOS activity, there was a sex difference in the OXPHOS activity, with male germ cells having higher activity than females. In addition, in the male fetal germ cells from E13.5 to E18.5, OXPHOS showed a decreasing trend while the glycolysis showed an increasing trend, indicating a shift in energy metabolism toward glycolysis for differentiation into spermatogonial stem cells, as described below. Whereas in female germ cells, OXPHOS exhibited no significant changes and glycolytic activity was increased, though the biological significance of this change has not been elucidated (Fig. 3, 4). In this context, we have evaluated the OXPHOS energy resources by measuring the oxidation of fatty acids, glutamine, and pyruvate using mitochondrial fuel flex analysis. As a result, while male gonocytes at E13.5 exhibited comparable dependence on these metabolites, female germ cells exhibited a greater dependence on fatty acid and pyruvate oxidation than males, suggesting that there is a higher expenditure of fatty acids and pyruvate in female germ cells [Hayashi et al., 2020] (Fig. 4).

To elucidate the sex differences other than energy metabolism in detail, we performed proteomic and metabolomic analyses of male and female germ cells at E13.5 and E18.5 [Hayashi et al., 2020]. In male germ cells, RNA processing, translation, and nucleotide synthesis are dominant in E13.5 and then decline until E18.5. In contrast, TCA cycle and Ser-Gly-one-carbon metabolism (SGOC), which includes serine and glycine biosynthesis, the folate cycle (which is critical for purine biosynthesis), and the methionine cycle, are consistently upregulated in fetal male germ cells. Interestingly, both pathways have been suggested to be closely related to epigenetic regulation through several metabolites including α-KG, FAD, succinate, and fumarate in the TCA cycle and SAM in SGOC [Haws et al., 2020], and their consistent enhancement may contribute to the regulation of epigenetics during male germ cell differentiation (Fig. 3). Related to these metabolisms, a study in rats showed that during the phase of mitotic arrest, male gonocytes undergo not only genome-wide DNA re-methylation, but also a pronounced introduction of H3K4me3 [Rwigemera et al., 2017]. To clarify the relationship between such epigenetic changes and metabolic regulation is one of the important future challenges. In this context, a recent study illustrated the importance of epigenome-metabolism crosstalk during mitotic arrest in male gonocytes. Inhibition of fatty acid oxidation has been revealed to specifically reduce the levels of H3K27ac in the gonads, presumably due to the decrease of acetyl-CoA production. This epigenetic change caused the downregulation of male differentiation-specific gene expression, and led to gonocytes exiting from mitotic arrest [Xu and Xie, 2021].

In females, increased protein stability and OXPHOS during fetal germ cell differentiation was shown, consistent with the observation that the number of mitochondria continues to increase during oogenesis [Jansen and de Boer, 1998; Hayashi et al., 2020]. These studies suggest a continuous upregulation of aerobic energy metabolism, which likely contributes to the proteostasis required for oocyte growth and maturation in subsequent stages. Further studies have shown that both pyruvate and fatty acid metabolism, both of which are important energy resources as described above, are essential for oocyte differentiation. The inhibition of pyruvate uptake to mitochondria by adding a mitochondrial pyruvate carrier (MPC) inhibitor, UK5099, to organ cultures of fetal mouse ovaries resulted in repressed early folliculogenesis without affecting survival of oocytes and meiosis [Tanaka et al., 2021]. The expression of the TGFβ-related genes *Gdf9* and *Bmp15* in ovarian germ cells, which are crucial for folliculogenesis, was downregulated by UK5099, suggesting that insufficient *Gdf9* expression in oocytes results in early follicular dysgenesis. Moreover, since addition of α-KG could rescue the effects of UK5099 in the ovarian culture, epigenetic regulation through the TCA cycle and α-KG might relate to the gene expression changes. On the other hand, the inhibition of fatty acid oxidation by the inhibitor of the key rate-limiting enzyme carnitine acyltransferase I (CPT1), etomoxir, resulted in a significant decrease of PGC numbers around E11.5–E13.5 in *in vitro* cultured fetal ovaries [Teng et al., 2016]. Treatment with etomoxir caused the increased expression of Ca^2+^/CamKII/5′-adenosine monophosphate-activated protein kinase (AMPK), phosphorylated AMPK, phosphorylated p53, and cyclin-dependent kinase inhibitor 1 (p21), suggesting that activation of the p53 pathway accounted for the reduced proliferation of gonadal germ cells. The decrease in ATP synthesis due to FAO inhibition seems to promote AMPK phosphorylation. In addition, the inhibition of FAO by etomoxir was also shown to significantly decrease the proportion of mature follicles, indicating that the metabolic pathway contributes to the subsequent folliculogenesis [Tanaka et al., 2021]. Thus, pyruvate and fatty acid metabolism not only contribute to energy supply in germ cells, but also to the regulation of fetal oocyte differentiation through specific signaling pathways (Fig. 4).

Epigenetic regulations in fetal oocyte differentiation have also been elucidate. For instance, a DNA demethylase TET1, which uses α-KG derived from TCA cycle as a co-factor, has an important role in regulating meiosis through 5mC DNA demethylation in a subset of meiotic genes [Yamaguchi et al., 2012], suggesting the relationship between meiosis and the TCA cycle metabolites. Another recent study revealed that maternal vitamin C, an essential micronutrient, is also required for proper DNA demethylation through TET1 and the development of female fetal germ cells in a mouse model. Maternal vitamin C deficiency does not affect overall embryonic development but leads to reduced numbers of germ cells, delayed meiosis, and reduced fecundity in adult offspring [DiTroia et al., 2019]. In addition, decreased expression of FAD-dependent histone demethylase LSD1 is involved in the programmed oocyte death by autophagy in perinatal mice through increased H3K4me2 and p62 expression [He et al., 2020], indicating the involvement of the TCA cycle regulation also in this process.

A recent paper described a remarkable characteristic of fetal germ cells related to the metabolic characteristics: the hyper-transcription/-translation [Percharde et al., 2017a, 2017b]. The hyper-transcription/-translation state of germ cells at around E13.5 compared with corresponding somatic cells is driven by the Myc (myelocytomatosis oncogene) factors n-Myc and l-Myc and by P-TEFb (positive transcription elongation factor b), a kinase complex that promotes transcriptional elongation via phosphorylation of serine 2 in the C-terminal domain of RNA polymerase II. This feature is consistent with our previous findings that the synthesis of biomass, including amino acids, nucleic acids, and glutathione are enhanced in E13.5 male gonocytes [Hayashi et al., 2017]. Interestingly, the extent of hyper-transcription/-translation of fetal germ cells is greater in males than in females similar to the OXPHOS activity, and it ceases in female germ cells around E15.5, earlier than in male germ cells. Although the physiologic significance of hyper-transcription/-translation in fetal germ cells is currently unclear, it appears to be correlated with the large size of germ cells [Percharde et al., 2017b]. All these findings suggested that fetal germ cells likely exhibit specific sex- and developmental stage-dependent metabolomic and proteomic characteristics.

In summary, several studies have reported the metabolic characteristics during fetal germ cell differentiation in gonads, while those in migrating and proliferating PGCs are hardly revealing. However, PGCs undergo extensive epigenetic reprogramming around this phase, including genome-wide 5mC DNA demethylation [Hajkova et al., 2002; Seisenberger et al., 2012]. In addition, the regulation of DNA demethylation by TET1, which is activated by α-KG and inhibited by succinate and fumarate, has been suggested to play an important role in PGC-to-gonocyte transition through the activation of germline reprogramming-responsive genes involved in gamete generation and meiosis [Hill et al., 2018]. These studies indicate that various types of metabolism-epigenome crosstalk may play an important role in appropriate epigenome remodeling and germ cell differentiation, even in migrating PGCs.

## Metabolism in Spermatogenesis

Germ cells in testes have peculiar nutritional requirements during spermatogenesis, switching their metabolic profile for energy production during their various developmental stages [Setchell, 2004; Rato et al., 2012]. Spermatogonia are supplied with nutrients from blood components and use glucose as fuel for ATP production (Fig. 3). Indeed, Myc/Mycn-mediated glycolysis has been reported to enhance mouse spermatogonial stem cell (SSC) self-renewal [Kanatsu-Shinohara et al., 2016]. However, aberrant glycolytic activation mediated by c-Jun N-terminal kinase (JNK) has also been shown to contribute to SSC aging [Kanatsu-Shinohara et al., 2019], indicating that the appropriate control of glycolysis is important for the maintenance of stemness in SSCs. Recently, single-cell RNA-seq analysis identified transitions from glycolytic to mitochondrial metabolism during mouse and human SSC differentiation [Guo et al., 2017; Hermann et al., 2018]. Spermatocytes are intermediate developing germ cells differentiated from spermatogonia and progress through meiosis. The single-cell RNA-seq analysis also illustrated enhanced expression of genes involved in OXPHOS and mitochondrial function among pachytene spermatocytes compared to spermatogonia. In contrast, an analysis of energy metabolic activities of rat spermatocytes and spermatids revealed that the activity of glycolytic enzymes was higher in spermatocytes than spermatids, while enzyme activities in the TCA cycle were higher in spermatids than spermatocytes [Bajpai et al., 1998]. Taken together, spermatocytes may depend on both glycolysis and OXPHOS (Fig. 3).

Energy metabolism in testicular germ cells after spermatogonia utilizes lactate as the central energy metabolite [Jutte et al., 1981]. This metabolite is produced by somatic Sertoli cells, transported to and used by germ cells [Boussouar and Benahmed, 2004]. Utilization of lactate by spermatocytes has also been reported, especially those that lie closer to the adluminal compartment [Rato et al., 2012]. In contrast, several studies have proposed that there is effective transport of long polyenes from Sertoli cells to germ cells [Rato et al., 2012], and pachytene spermatocytes actively metabolize fatty acids [Retterstøl et al., 2001]. In addition, network modeling analysis of metabolic pathways activated in mouse spermatogenesis inferred that the most active pathway during spermatogonial differentiation and meiotic initiation was the TCA cycle, and that its energy source was fatty acid oxidation [Whitmore and Ye, 2015]. Therefore, lactate and fatty acids would be the best candidates for energy sources in spermatocytes. Round spermatids have been shown to exhibit lower glycolytic potential than testicular germ cells at earlier developmental stages, and energy metabolism of spermatids is mainly dependent on lactate present in extracellular medium and supplied by Sertoli cells [Boussouar and Benahmed, 2004; Rato et al., 2012]. On the other hand, spermatozoa showed significantly higher activity of glycolytic enzymes than those in other testicular germ cells, using only glucose or fructose for their energy metabolism [Bajpai et al., 1998]. Furthermore, energy metabolism in spermatozoa is compartmentalized so that mitochondria and OXPHOS are restricted to the midpiece, while glycolysis occurs in the principal piece [Rato et al., 2012] (Fig. 3).

Our knowledge of the metabolic characteristics other than energy metabolism in spermatogenesis is rather limited. The pentose phosphate pathway (PPP), which is involved in the nucleotide synthesis and reactive oxygen species (ROS) metabolism through the generation of pentoses and NADPH, respectively, has been reported to be more active in spermatocytes than in spermatids and spermatozoa, though the biological significance of this pathway in spermatocytes has not been clarified [Bajpai et al., 1998]. On the other hand, several analyses have been shown that deficiency of one-carbon metabolism is also associated with male infertility [Singh and Jaiswal, 2013]. The male knockout mouse of an enzyme in the pathway, methylene tetrahydrofolate reductase (MTHFR), which converts 5,10-methylene tetrahydrofolate (THF) to 5-methyl THF and promotes the conversion of homocysteine to methionine, showed high homocysteine levels, reduced germ cell counts, impaired spermatogenesis, and infertility [Frosst et al., 1995]. A further study of MTHFR deficiency in early germ cell development found different reproductive phenotypes between 2 strains, BALB/c mice and C57BL/6 mice: BALB/c mice showed an early postnatal loss of germ cell number and proliferation that resulted in infertility with perturbed DNA methylation patterns of imprinted genes in sperms, whereas the C57BL/6 mice exhibited decreased sperm numbers and altered testicular histology but showed normal fertility and unaffected imprinting [Chan et al., 2010]. Although it has been suggested that other enzymes in the folate cycle may have strain-specific compensatory effects, the cause of phenotypic differences between strains has not been identified. In addition, the C677T *MTHFR* variation has been reported to be associated with decreased enzymatic activity, and several polymorphism studies have revealed that the homo (TT) genotype mutant of C677T *MTHFR* gene showed higher prevalence in infertile men [Singh and Jaiswal, 2013].

The process of gametogenesis in male adults has also been shown to involve diverse changes in the epigenome, though relationship with metabolic status are largely unknown. Male gametogenesis occurs without significant changes in 5mC DNA methylation and instead involves transcription of promoters bearing high RNA polymerase II, H3K9 acetylation (H3K9ac), H3K4me3, low CG content, and (often) 5-hydroxymethylcytosine (5hmC) [Hammoud et al., 2014]. Also, establishment of bivalent domains occurs during the transition from differentiating spermatogonia to pachytene spermatocytes via acquisition of H3K27me3 by the recruitment of polycomb repressive complex 2 (PRC2) through germline-specific Polycomb protein, SCML2 [Maezawa et al., 2018]. This suggests the possibility that SAM supply through one-carbon metabolism is required for H3K27me3 by enhancer of zeste homolog (EZH)-type HMTs, the enzymatic components of PRC2, in the spermatogonia-to-spermatocyte transition. Furthermore, a recent study revealed that TET1 was required for the maintenance of SSCs with age through 5mC DNA demethylation and gene expression related to meiosis and spermatogenesis [Huang et al., 2020], suggesting the involvement of various metabolites related to TET1 in this process.

## Metabolism in Oogenesis

During the growth phase, oocytes accumulate RNAs, proteins, and organelles as maternal factors required in early cleavage-stage embryos [Li et al., 2010], and mitochondrial copy number per germ cell was reported to be continuously increased [Jansen and de Boer, 1998]. Throughout the growth period, pyruvate and oxygen consumption is also gradually increased in mouse oocytes [Harris et al., 2009; Gu et al., 2015]. These studies suggest that unlike spermatogenesis, energy metabolism in oocyte growth is consistently dependent on OXPHOS (Fig. 4). Once oocytes reach the fully-grown germinal vesicle (GV) stage, mRNA synthesis ceases and subsequent maturation-inducing hormone stimulates the resumption of meiosis, characterized by GV breakdown (GVBD). Oocytes then proceed through the meiosis I (MI) division and are arrested at metaphase II (MII) until fertilization [Wang and Sun, 2007]. The fully-grown oocytes also have a limited capacity for glucose metabolism [Biggers et al., 1967; Saito et al., 1994], so glucose needs to be metabolized into pyruvate by follicular cells and then transported into oocytes to progress meiotic maturation successfully [Leese and Barton, 1985; Downs and Mastropolo, 1994; Leese, 2015]. The importance of pyruvate metabolism in oocytes during maturation has also been proven using oocyte-specific knockout mice of *Pdha1* (pyruvate dehydrogenase E1 a), which caused severe meiotic defects [Johnson et al., 2007]. *Pdha1*-knockout oocytes also exhibited reduced levels of ATP and NAD(P)H compared with wild-type oocytes, indicating the dysfunctional energy metabolism. A growing number of reports have also shown that fatty acid oxidation plays a critical role in oocyte meiotic resumption and developmental potential [Ferguson and Leese, 2006; Downs et al., 2009; Dunning et al., 2010]. Taken together, OXPHOS, mainly using pyruvate and fatty acids as resources, is critical for the energy supply during oocyte growth and maturation (Fig. 4).

Recent studies have revealed the metabolomic and proteomic characteristics of oocytes during the growth and maturation process [Peñalver Bernabé et al., 2019; Li et al., 2020]. A comprehensive metabolomic analysis during postnatal oocyte growth demonstrated stage-dependent metabolic characteristics in ovarian follicles [Peñalver Bernabé et al., 2019]. For instance, enrichment analysis of metabolic pathways in each stage revealed that growing oocytes have increased starch and sucrose metabolism, while oocytes in the primordial follicles specifically have enriched proline and arginine metabolism. In contrast, the significant metabolic pathways in oocytes in primary, secondary, and antral follicles were CoA catabolism and fatty acid oxidation in the peroxisome, suggesting that acetyl-CoA production was enhanced, which may cause the increase of histone acetylation in these stages of follicles. Furthermore, an analysis of key metabolites and genes during oocyte growth revealed the high levels of nitric oxide and NOS (nitric oxide synthase) in primordial oocytes and metabolites involved in the PPP in oocytes during the primary and later follicle stages [Peñalver Bernabé et al., 2019]. Though the functional significance of PPP in primary and later follicle stages has not been elucidated, PPP-mediated nucleotide biosynthesis may be required for oocyte growth. A further study revealed that NOS in primordial oocytes and pre-granulosa cells synthesized NO, which activates cGMP-PKG signaling and subsequently downregulates an E3 ubiquitin ligase, F-box and WD repeat domain containing 7, FBXW7, resulting in the stabilization of mTOR in pre-granulosa cells [Zhao et al., 2020]. This process activates KITL expression in pre-granulosa cells, triggers the transduction of KITL/KIT/PI3K/AKT/FOXO3a signaling in oocytes, and consequently promotes primordial follicule activation, oocyte growth, and granulosa cell proliferation in neonatal ovaries.

A more recent analysis demonstrated the characteristic metabolic pathways during oocyte maturation, in which amino acids, carbohydrates, and nucleotides increased during meiotic resumption and/or maturation, whereas lipid metabolites displayed a notable reduction during meiotic resumption (Fig. 4) [Li et al., 2020]. Among these observations, the authors showed that a decrease in polyunsaturated fatty acids (PUFAs) was important for the resumption of meiosis. GV oocytes cultured in medium supplemented with one of PUFAs, arachidonic acid (ARA), resulted in the meiotic defects and decreased accumulation of NFKB activating protein (NKAP). Since NKAP showed a function in the regulation of the meiotic apparatus, the fall in PUFAs during meiotic resumption allows NKAP to appropriately control the meiotic progression. They also indicated that the SGOC pathway was upregulated during meiotic resumption, and knockdown of a related enzyme, serine hydroxymethyltransferase 2 (SHMT2), disturbed the epigenetic profile during oocyte maturation including the decrease of 5mC DNA methylation and H3K4me3, resulting in the perturbation of embryonic development after fertilization. They also demonstrated the importance of carbohydrate metabolism related to the TCA cycle and PPP during oocyte maturation. Related to PPP, the knockdown of the rate-limiting enzyme, glucose-6-phosphate dehydrogenase (G6PD), resulted in excessive ROS production and decreased maturation rate, indicating the role in ROS metabolism.

## Dietary Interventions for Reproductive Function

It is recognized that sperm and eggs are greatly influenced by the nutritional environment, some of which are known to affect intracellular metabolism and the epigenome [Hunt and Hassold, 2008; Nassan et al., 2018]. For instance, folate is a key source of the one-carbon metabolism involved in DNA and histone methylation [Crider et al., 2012]. Maternal folate deficiency during pregnancy and lactation in mice resulted in aberrant DNA methylation patterns in the offspring's sperm, whereas chronic paternal folate deficiency was associated with decreased pregnancy rates [Lambrot et al., 2013]. In addition, antioxidants and omega-3 fatty acids have been cited as essential nutrients for producing healthy sperm with high fertility [Nassan et al., 2018]. Furthermore, several studies reported an improvement of human sperm quality through dietary supplementation with vitamin C, vitamin D, vitamin E, β-carotene, lycopene, and zinc [Schagdarsurengin and Steger, 2016] (Fig. 3). Among such nutrients, vitamin D has also been found to affect sperm epigenetics through the interaction with the vitamin D_3_ receptor, histone acetyltransferases, and other chromatin modifiers [Karlic and Varga, 2011].

Obesity has also been found to associate with male and female infertility [Schagdarsurengin and Steger, 2016; Broughton and Moley, 2017]. Obesity is well known to increase oxidative stress through excess ROS production by augmented expression of NADPH oxidase and decreased expression of antioxidative enzymes [Furukawa et al., 2004]. Such perturbation of ROS metabolism has been elucidated to cause sperm DNA fragmentation, resulting in reduced sperm quality and *de novo* mutations in the embryo [Eisenberg et al., 2014; Schagdarsurengin and Steger, 2016]. A review reported that antioxidant supplementation for subfertile men can improve both clinical pregnancy rates and live birth rates [Showell et al., 2020]. Moreover, improvements in sperm count, morphology, and motility have been observed following weight loss in both men and mice [Håkonsen et al., 2011; Palmer et al., 2012]. Since male obese mice showed aberrant DNA methylation and miRNA expression patterns in their sperm, obesity-induced male subfertility might also be caused by epigenetic changes other than oxidative stress [Fullston et al., 2012]. In this context, male mice fed with high-fat diets showed decreased expression of histone deacetylase SIRT6 in elongating spermatids, resulting in increased histone acetylation [Palmer et al., 2011]. It is also possible that excess fat may affect the epigenome by increasing fatty acid metabolism and promoting the synthesis of acetyl-CoA in obese mice.

As for dietary interventions other than high-fat diets, a low-protein diet in male mice decreases H3K27me3 and increases DNA promoter methylation at *Ppara* in the liver of offspring [Carone et al., 2010]. A more recent study reported that a low-protein diet induces ATF7 activation via ROS in testes, leading to H3K9me2 reduction in ATF7 target genes. This epigenetic change is maintained in spermatozoa and affects the pattern of small RNA expression [Yoshida et al., 2020]. All these studies suggest that dietary and supplemental interventions strongly influence reproductive function, which may also be important in fetal germ cell formation and differentiation.

Obese women are more likely to have ovulatory dysfunction and demonstrate poorer outcomes with the use of *in vitro* fertilization [Broughton and Moley, 2017]. Obesity appears to affect oocytes through disrupted meiotic spindle formation and mitochondrial dynamics. The recent comparative proteomic analysis revealed the reduced expression of TIGAR (TP53-induced glycolysis and apoptosis regulator) protein in ovulated oocytes from obese mice, promoting the ROS accumulation and meiotic defects [Wang et al., 2018]. Moreover, excess free fatty acids might have a toxic effect on oocytes, which damage non-adipose cells by increasing ROS to induce mitochondrial and endoplasmic reticulum (ER) stress leading to apoptosis [Igosheva et al., 2010; Jungheim et al., 2011]. A recent study reported that obesity induces the loss of Stella, a maternal factor required for the protection of 5mC DNA methylation from TET3-mediated demethylation by binding to maternal chromatin containing H3K9me2 [Nakamura et al., 2012], and causes rapid conversion of 5mC to 5hmC in the maternal pronuclei of zygotes, thereby contributing to reproductive disorders, though the molecular mechanism of Stella regulation in obese mice has not been elucidated [Han et al., 2018].

## Conclusion

In recent years, the development of omics technologies has enabled comprehensive metabolic analysis in a relatively small number of cells, and the analysis of metabolic characteristics of germ cells is progressing. However, comprehensive metabolic analysis in early PGCs and testicular germ cells has not yet been performed and requires clarification. It is also an important future task to elucidate how the metabolic pathways characteristic in germ cells at each stage are involved in the regulation of germ cell function and differentiation, especially through epigenetic regulation. Such research would lead to suggestions for optimal diets and supplements to maintain reproductive health through modulating metabolic and epigenetic status. Furthermore, elucidating the mechanisms that influence the phenotype of the next generation through metabolism-epigenome crosstalk in germ cells is expected to lead to an understanding of molecular mechanisms of epigenetic inheritance and to innovations in assisted reproductive technology through the construction of a culture environment to maintain proper metabolism and epigenome.

## Conflict of Interest Statement

The authors have no conflicts of interest to declare.

## Funding Sources

For part of this work, Y.H. was supported by Grant-in-Aid for Scientific Research (KAKENHI) (C) (grant 19K06434), KAKENHI in the Innovative Areas, “Sex Spectrum” (grant 20H04917) and “Ensuring integrity in gametogenesis” (grant 19H05238) from the Ministry of Education, Culture, Sports, Science and Technology of Japan (MEXT), Takeda Science Foundation, Kato Memorial Bioscience Foundation, the Inamori Foundation, and Astellas Foundation for Research on Metabolic Disorders. Y.M. was supported by KAKENHI (B) (grant 19H03231) from MEXT and The Uehara Memorial Foundation.

## Author Contributions

Y.H. designed and prepared the manuscript. Y.M. helped in the design and critical discussions.

## References

[B1] Agathocleous M, Harris WA (2013). Metabolism in physiological cell proliferation and differentiation. Trends Cell Biol.

[B2] Anand R, Marmorstein R (2007). Structure and mechanism of lysine-specific demethylase enzymes. J Biol Chem.

[B3] Bajpai M, Gupta G, Setty BS (1998). Changes in carbohydrate metabolism of testicular germ cells during meiosis in the rat. Eur J Endocrinol.

[B4] Biggers JD, Whittingham DG, Donahue RP (1967). The pattern of energy metabolism in the mouse oöcyte and zygote. Proc Natl Acad Sci U S A.

[B5] Boussouar F, Benahmed M (2004). Lactate and energy metabolism in male germ cells. Trends Endocrinol Metab.

[B6] Brinster RL, Harstad H (1977). Energy metabolism in primordial germ cells of the mouse. Exp Cell Res.

[B7] Broughton DE, Moley KH (2017). Obesity and female infertility: potential mediators of obesity's impact. Fertil Steril.

[B8] Carey BW, Finley LW, Cross JR, Allis CD, Thompson CB (2015). Intracellular α-ketoglutarate maintains the pluripotency of embryonic stem cells. Nature.

[B9] Carone BR, Fauquier L, Habib N, Shea JM, Hart CE, Li R (2010). Paternally induced transgenerational environmental reprogramming of metabolic gene expression in mammals. Cell.

[B10] Chakrabarty RP, Chandel NS (2021). Mitochondria as signaling organelles control mammalian stem cell fate. Cell Stem Cell.

[B11] Chan D, Cushnie DW, Neaga OR, Lawrance AK, Rozen R, Trasler JM (2010). Strain-specific defects in testicular development and sperm epigenetic patterns in 5,10-methylenetetrahydrofolate reductase-deficient mice. Endocrinology.

[B12] Crider KS, Yang TP, Berry RJ, Bailey LB (2012). Folate and DNA methylation: a review of molecular mechanisms and the evidence for folate's role. Adv Nutr.

[B13] Dang W, Steffen KK, Perry R, Dorsey JA, Johnson FB, Shilatifard A (2009). Histone H4 lysine 16 acetylation regulates cellular lifespan. Nature.

[B14] DiTroia SP, Percharde M, Guerquin MJ, Wall E, Collignon E, Ebata KT (2019). Maternal vitamin C regulates reprogramming of DNA methylation and germline development. Nature.

[B15] Doi M, Hirayama J, Sassone-Corsi P (2006). Circadian regulator CLOCK is a histone acetyltransferase. Cell.

[B16] Downs SM, Mastropolo AM (1994). The participation of energy substrates in the control of meiotic maturation in murine oocytes. Dev Biol.

[B17] Downs SM, Mosey JL, Klinger J (2009). Fatty acid oxidation and meiotic resumption in mouse oocytes. Mol Reprod Dev.

[B18] Dunning KR, Cashman K, Russell DL, Thompson JG, Norman RJ, Robker RL (2010). Beta-oxidation is essential for mouse oocyte developmental competence and early embryo development. Biol Reprod.

[B19] Eisenberg ML, Kim S, Chen Z, Sundaram R, Schisterman EF, Buck Louis GM (2014). The relationship between male BMI and waist circumference on semen quality: data from the LIFE study. Hum Reprod.

[B20] Fan J, Krautkramer KA, Feldman JL, Denu JM (2015). Metabolic regulation of histone post-translational modifications. ACS Chem Biol.

[B21] Ferguson EM, Leese HJ (2006). A potential role for triglyceride as an energy source during bovine oocyte maturation and early embryo development. Mol Reprod Dev.

[B22] Feldman JL, Dittenhafer-Reed KE, Denu JM (2012). Sirtuin catalysis and regulation. J Biol Chem.

[B23] Floros VI, Pyle A, Dietmann S, Wei W, Tang WCW, Irie N (2018). Segregation of mitochondrial DNA heteroplasmy through a developmental genetic bottleneck in human embryos. Nat Cell Biol.

[B24] Frosst P, Blom HJ, Milos R, Goyette P, Sheppard CA, Matthews RG (1995). A candidate genetic risk factor for vascular disease: a common mutation in methylenetetrahydrofolate reductase. Nat Genet.

[B25] Fullston T, Palmer NO, Owens JA, Mitchell M, Bakos HW, Lane M (2012). Diet-induced paternal obesity in the absence of diabetes diminishes the reproductive health of two subsequent generations of mice. Hum Reprod.

[B26] Furukawa S, Fujita T, Shimabukuro M, Iwaki M, Yamada Y, Nakajima Y (2004). Increased oxidative stress in obesity and its impact on metabolic syndrome. J Clin Invest.

[B27] Ginsburg M, Snow MH, McLaren A (1990). Primordial germ cells in the mouse embryo during gastrulation. Development.

[B28] Gu L, Liu H, Gu X, Boots C, Moley KH, Wang Q (2015). Metabolic control of oocyte development: linking maternal nutrition and reproductive outcomes. Cell Mol Life Sci.

[B29] Guo J, Grow EJ, Yi C, Mlcochova H, Maher GJ, Lindskog C (2017). Chromatin and Single-Cell RNA-Seq Profiling Reveal Dynamic Signaling and Metabolic Transitions during Human Spermatogonial Stem Cell Development. Cell Stem Cell.

[B30] Hajkova P, Erhardt S, Lane N, Haaf T, El-Maarri O, Reik W (2002). Epigenetic reprogramming in mouse primordial germ cells. Mech Dev.

[B31] Håkonsen LB, Thulstrup AM, Aggerholm AS, Olsen J, Bonde JP, Andersen CY (2011). Does weight loss improve semen quality and reproductive hormones? Results from a cohort of severely obese men. Reprod Health.

[B32] Hammoud SS, Low DH, Yi C, Carrell DT, Guccione E, Cairns BR (2014). Chromatin and transcription transitions of mammalian adult germline stem cells and spermatogenesis. Cell Stem Cell.

[B33] Han L, Ren C, Li L, Li X, Ge J, Wang H (2018). Embryonic defects induced by maternal obesity in mice derive from Stella insufficiency in oocytes. Nat Genet.

[B34] Hanna CW, Demond H, Kelsey G (2018). Epigenetic regulation in development: is the mouse a good model for the human?. Hum Reprod Update.

[B35] Harris SE, Leese HJ, Gosden RG, Picton HM (2009). Pyruvate and oxygen consumption throughout the growth and development of murine oocytes. Mol Reprod Dev.

[B36] Haws SA, Leech CM, Denu JM (2020). Metabolism and the Epigenome: A Dynamic Relationship. Trends Biochem Sci.

[B37] Hayashi K, Ohta H, Kurimoto K, Aramaki S, Saitou M (2011). Reconstitution of the mouse germ cell specification pathway in culture by pluripotent stem cells. Cell.

[B38] Hayashi Y, Otsuka K, Ebina M, Igarashi K, Takehara A, Matsumoto M (2017). Distinct requirements for energy metabolism in mouse primordial germ cells and their reprogramming to embryonic germ cells. Proc Natl Acad Sci U S A.

[B39] Hayashi Y, Mori M, Igarashi K, Tanaka K, Takehara A, Ito-Matsuoka Y (2020). Proteomic and metabolomic analyses uncover sex-specific regulatory pathways in mouse fetal germline differentiation. Biol Reprod.

[B40] He M, Zhang T, Zhu Z, Qin S, Wang H, Zhao L (2020). LSD1 contributes to programmed oocyte death by regulating the transcription of autophagy adaptor SQSTM1/p62. Aging Cell.

[B41] Hermann BP, Cheng K, Singh A, Roa-De La Cruz L, Mutoji KN, Chen IC (2018). The Mammalian Spermatogenesis Single-Cell Transcriptome, from Spermatogonial Stem Cells to Spermatids. Cell Rep.

[B42] Hill PWS, Leitch HG, Requena CE, Sun Z, Amouroux R, Roman-Trufero M (2018). Epigenetic reprogramming enables the transition from primordial germ cell to gonocyte. Nature.

[B43] Huang G, Liu L, Wang H, Gou M, Gong P, Tian C (2020). Tet1 Deficiency Leads to Premature Reproductive Aging by Reducing Spermatogonia Stem Cells and Germ Cell Differentiation. iScience.

[B44] Hunt PA, Hassold TJ (2008). Human female meiosis: what makes a good egg go bad?. Trends Genet.

[B45] Igosheva N, Abramov AY, Poston L, Eckert JJ, Fleming TP, Duchen MR (2010). Maternal diet-induced obesity alters mitochondrial activity and redox status in mouse oocytes and zygotes. PLoS One.

[B46] Irie N, Weinberger L, Tang WW, Kobayashi T, Viukov S, Manor YS (2015). SOX17 is a critical specifier of human primordial germ cell fate. Cell.

[B47] Ito S, Shen L, Dai Q, Wu SC, Collins LB, Swenberg JA (2011). Tet proteins can convert 5-methylcytosine to 5-formylcytosine and 5-carboxylcytosine. Science.

[B48] Jansen RP, de Boer K (1998). The bottleneck: mitochondrial imperatives in oogenesis and ovarian follicular fate. Mol Cell Endocrinol.

[B49] Johnson MT, Freeman EA, Gardner DK, Hunt PA (2007). Oxidative metabolism of pyruvate is required for meiotic maturation of murine oocytes in vivo. Biol Reprod.

[B50] Jungheim ES, Macones GA, Odem RR, Patterson BW, Lanzendorf SE, Ratts VS (2011). Associations between free fatty acids, cumulus oocyte complex morphology and ovarian function during in vitro fertilization. Fertil Steril.

[B51] Jutte NH, Grootegoed JA, Rommerts FF, van der Molen HJ (1981). Exogenous lactate is essential for metabolic activities in isolated rat spermatocytes and spermatids. J Reprod Fertil.

[B52] Kanatsu-Shinohara M, Tanaka T, Ogonuki N, Ogura A, Morimoto H, Cheng PF (2016). Myc/Mycn-mediated glycolysis enhances mouse spermatogonial stem cell self-renewal. Genes Dev.

[B53] Kanatsu-Shinohara M, Yamamoto T, Toh H, Kazuki Y, Kazuki K, Imoto J (2019). Aging of spermatogonial stem cells by Jnk-mediated glycolysis activation. Proc Natl Acad Sci U S A.

[B54] Karlic H, Varga F (2011). Impact of vitamin D metabolism on clinical epigenetics. Clin Epigenetics.

[B55] Kurimoto K, Yabuta Y, Hayashi K, Ohta H, Kiyonari H, Mitani T (2015). Quantitative Dynamics of Chromatin Remodeling during Germ Cell Specification from Mouse Embryonic Stem Cells. Cell Stem Cell.

[B56] Krishnaiah SY, Wu G, Altman BJ, Growe J, Rhoades SD, Coldren F (2017). Clock Regulation of Metabolites Reveals Coupling between Transcription and Metabolism. Cell Metab.

[B57] Lambrot R, Xu C, Saint-Phar S, Chountalos G, Cohen T, Paquet M (2013). Low paternal dietary folate alters the mouse sperm epigenome and is associated with negative pregnancy outcomes. Nat Commun.

[B58] Lee Chong T, Ahearn EL, Cimmino L (2019). Reprogramming the epigenome with vitamin C. Front Cell Dev Biol.

[B59] Leese HJ (2015). History of oocyte and embryo metabolism. Reprod Fertil Dev.

[B60] Leese HJ, Barton AM (1985). Production of pyruvate by isolated mouse cumulus cells. J Exp Zool.

[B61] Lei L, Spradling AC (2013). Mouse primordial germ cells produce cysts that partially fragment prior to meiosis. Development.

[B62] Li L, Zheng P, Dean J (2010). Maternal control of early mouse development. Development.

[B63] Li L, Zhu S, Shu W, Guo Y, Guan Y, Zeng J (2020). Characterization of Metabolic Patterns in Mouse Oocytes during Meiotic Maturation. Mol Cell.

[B64] Li Z, Fang F, Zhao Q, Li H, Xiong C (2019). Supplementation of vitamin C promotes early germ cell specification from human embryonic stem cells. Stem Cell Res Ther.

[B65] Liberti MV, Locasale JW (2016). The Warburg Effect: How Does it Benefit Cancer Cells?. Trends Biochem Sci.

[B66] Maezawa S, Hasegawa K, Yukawa M, Kubo N, Sakashita A, Alavattam KG (2018). Polycomb protein SCML2 facilitates H3K27me3 to establish bivalent domains in the male germline. Proc Natl Acad Sci U S A.

[B67] Matsui Y, Mochizuki K (2014). A current view of the epigenome in mouse primordial germ cells. Mol Reprod Dev.

[B68] Nakahata Y, Kaluzova M, Grimaldi B, Sahar S, Hirayama J, Chen D (2008). The NAD+-dependent deacetylase SIRT1 modulates CLOCK-mediated chromatin remodeling and circadian control. Cell.

[B69] Nakamura T, Liu YJ, Nakashima H, Umehara H, Inoue K, Matoba S (2012). PGC7 binds histone H3K9me2 to protect against conversion of 5mC to 5hmC in early embryos. Nature.

[B70] Nassan FL, Chavarro JE, Tanrikut C (2018). Diet and men's fertility: does diet affect sperm quality?. Fertil Steril.

[B71] Nichols J, Smith A (2009). Naive and primed pluripotent states. Cell Stem Cell.

[B72] Palmer NO, Fullston T, Mitchell M, Setchell BP, Lane M (2011). SIRT6 in mouse spermatogenesis is modulated by diet-induced obesity. Reprod Fertil Dev.

[B73] Palmer NO, Bakos HW, Owens JA, Setchell BP, Lane M (2012). Diet and exercise in an obese mouse fed a high-fat diet improve metabolic health and reverse perturbed sperm function. Am J Physiol Endocrinol Metab.

[B74] Peleg S, Feller C, Forne I, Schiller E, Sévin DC, Schauer T (2016). Life span extension by targeting a link between metabolism and histone acetylation in *Drosophila*. EMBO Rep.

[B75] Peñalver Bernabé B, Thiele I, Galdones E, Siletz A, Chandrasekaran S, Woodruff TK (2019). Dynamic genome-scale cell-specific metabolic models reveal novel inter-cellular and intra-cellular metabolic communications during ovarian follicle development. BMC Bioinformatics.

[B76] Percharde M, Bulut-Karslioglu A, Ramalho-Santos M (2017a). Hypertranscription in Development, Stem Cells, and Regeneration. Dev Cell.

[B77] Percharde M, Wong P, Ramalho-Santos M (2017b). Global Hypertranscription in the Mouse Embryonic Germline. Cell Rep.

[B78] Rato L, Alves MG, Socorro S, Duarte AI, Cavaco JE, Oliveira PF (2012). Metabolic regulation is important for spermatogenesis. Nat Rev Urol.

[B79] Retterstøl K, Haugen TB, Tran TN, Christophersen BO (2001). Studies on the metabolism of essential fatty acids in isolated human testicular cells. Reproduction.

[B80] Rwigemera A, Joao F, Delbes G (2017). Fetal testis organ culture reproduces the dynamics of epigenetic reprogramming in rat gonocytes. Epigenetics Chromatin.

[B81] Sabari BR, Zhang D, Allis CD, Zhao Y (2017). Metabolic regulation of gene expression through histone acylations. Nat Rev Mol Cell Biol.

[B82] Saitou M, Yamaji M (2012). Primordial germ cells in mice. Cold Spring Harb Perspect Biol.

[B83] Saito T, Hiroi M, Kato T (1994). Development of glucose utilization studied in single oocytes and preimplantation embryos from mice. Biol Reprod.

[B84] Sanderson SM, Gao X, Dai Z, Locasale JW (2019). Methionine metabolism in health and cancer: a nexus of diet and precision medicine. Nat Rev Cancer.

[B85] Sasaki H, Matsui Y (2008). Epigenetic events in mammalian germ-cell development: reprogramming and beyond. Nat Rev Genet.

[B86] Schagdarsurengin U, Steger K (2016a). Epigenetics in male reproduction: effect of paternal diet on sperm quality and offspring health. Nat Rev Urol.

[B87] Seisenberger S, Andrews S, Krueger F, Arand J, Walter J, Santos F (2012). The dynamics of genome-wide DNA methylation reprogramming in mouse primordial germ cells. Mol Cell.

[B88] Seki Y, Hayashi K, Itoh K, Mizugaki M, Saitou M, Matsui Y (2005). Extensive and orderly reprogramming of genome-wide chromatin modifications associated with specification and early development of germ cells in mice. Dev Biol.

[B89] Seki Y, Yamaji M, Yabuta Y, Sano M, Shigeta M, Matsui Y (2007). Cellular dynamics associated with the genome-wide epigenetic reprogramming in migrating primordial germ cells in mice. Development.

[B90] Setchell BP (2004). Hormones: what the testis really sees. Reprod Fertil Dev.

[B91] Shi Y, Lan F, Matson C, Mulligan P, Whetstine JR, Cole PA (2004). Histone demethylation mediated by the nuclear amine oxidase homolog LSD1. Cell.

[B92] Shirane K, Kurimoto K, Yabuta Y, Yamaji M, Satoh J, Ito S (2016). Global Landscape and Regulatory Principles of DNA Methylation Reprogramming for Germ Cell Specification by Mouse Pluripotent Stem Cells. Dev Cell.

[B93] Showell MG, Mackenzie-Proctor R, Jordan V, Hart RJ (2020). Antioxidants for female subfertility. Cochrane Database Syst Rev.

[B94] Singh K, Jaiswal D (2013). One-carbon metabolism, spermatogenesis, and male infertility. Reprod Sci.

[B95] Sivanand S, Viney I, Wellen KE (2018). Spatiotemporal Control of Acetyl-CoA Metabolism in Chromatin Regulation. Trends Biochem Sci.

[B96] Spradling A, Fuller MT, Braun RE, Yoshida S (2011). Germline stem cells. Cold Spring Harb Perspect Biol.

[B97] Sutton-McDowall ML, Gilchrist RB, Thompson JG (2010). The pivotal role of glucose metabolism in determining oocyte developmental competence. Reproduction.

[B98] Suzuki H, Kanai-Azuma M, Kanai Y (2015). From Sex Determination to Initial Folliculogenesis in Mammalian Ovaries: Morphogenetic Waves along the Anteroposterior and Dorsoventral Axes. Sex Dev.

[B99] Tanaka K, Hayashi Y, Takehara A, Ito-Matsuoka Y, Tachibana M, Yaegashi N (2021). Abnormal early folliculogenesis due to impeded pyruvate metabolism in mouse oocytes. Biol Reprod.

[B100] Teng H, Sui X, Zhou C, Shen C, Yang Y, Zhang P (2016). Fatty acid degradation plays an essential role in proliferation of mouse female primordial germ cells via the p53-dependent cell cycle regulation. Cell Cycle.

[B101] Thompson JG (2006). The impact of nutrition of the cumulus oocyte complex and embryo on subsequent development in ruminants. J Reprod Dev.

[B102] Tischler J, Gruhn WH, Reid J, Allgeyer E, Buettner F, Marr C (2019). Metabolic regulation of pluripotency and germ cell fate through α-ketoglutarate. EMBO J.

[B103] Tomizawa S, Nowacka-Woszuk J, Kelsey G (2012). DNA methylation establishment during oocyte growth: mechanisms and significance. Int J Dev Biol.

[B104] Tsogtbaatar E, Landin C, Minter-Dykhouse K, Folmes CDL (2020). Energy Metabolism Regulates Stem Cell Pluripotency. Front Cell Dev Biol.

[B105] Verdikt R, Allard P (2021). Metabolo-epigenetics: the interplay of metabolism and epigenetics during early germ cells development. Biol Reprod.

[B106] Verdin E (2015). NAD⁺ in aging, metabolism, and neurodegeneration. Science.

[B107] Wang H, Cheng Q, Li X, Hu F, Han L, Zhang H (2018). Loss of TIGAR Induces Oxidative Stress and Meiotic Defects in Oocytes from Obese Mice. Mol Cell Proteomics.

[B108] Wang Q, Sun QY (2007). Evaluation of oocyte quality: morphological, cellular and molecular predictors. Reprod Fertil Dev.

[B109] Warburg O (1956). On the origin of cancer cells. Science.

[B110] Weinberger L, Ayyash M, Novershtern N, Hanna JH (2016). Dynamic stem cell states: naive to primed pluripotency in rodents and humans. Nat Rev Mol Cell Biol.

[B111] Whitmore LS, Ye P (2015). Dissecting Germ Cell Metabolism through Network Modeling. PLoS One.

[B112] Wu X, Zhang Y (2017). TET-mediated active DNA demethylation: mechanism, function and beyond. Nat Rev Genet.

[B113] Xiao M, Yang H, Xu W, Ma S, Lin H, Zhu H (2012). Inhibition of α-KG-dependent histone and DNA demethylases by fumarate and succinate that are accumulated in mutations of FH and SDH tumor suppressors. Genes Dev.

[B114] Xing M, Wang N, Zeng H, Zhang J (2020). α-ketoglutarate promotes the specialization of primordial germ cell-like cells through regulating epigenetic reprogramming. J Biomed Res.

[B115] Xu W, Yang H, Liu Y, Yang Y, Wang P, Kim SH (2011). Oncometabolite 2-hydroxyglutarate is a competitive inhibitor of α-ketoglutarate-dependent dioxygenases. Cancer Cell.

[B116] Xu Y, Xie J (2021). Etomoxir regulates the differentiation of male germ cells by specifically reducing H3K27ac level. BMC Dev Biol.

[B117] Yamaguchi S, Hong K, Liu R, Shen L, Inoue A, Diep D (2012). Tet1 controls meiosis by regulating meiotic gene expression. Nature.

[B118] Ying QL, Wray J, Nichols J, Batlle-Morera L, Doble B, Woodgett J (2008). The ground state of embryonic stem cell self-renewal. Nature.

[B119] Yoshida K, Maekawa T, Ly NH, Fujita SI, Muratani M, Ando M (2020). ATF7-Dependent Epigenetic Changes Are Required for the Intergenerational Effect of a Paternal Low-Protein Diet. Mol Cell.

[B120] Zhao P, Song Z, Wang Y, Cai H, Du X, Li C (2020). The endothelial nitric oxide synthase/cyclic guanosine monophosphate/protein kinase G pathway activates primordial follicles. Aging (Albany NY).

